# Mental and physical health-related quality of life in patients with recurrent patellar dislocations—a generic and disease-specific quality of life questionnaire assessment

**DOI:** 10.1186/s40634-022-00499-3

**Published:** 2022-06-28

**Authors:** Peter Balcarek, Danko Dan Milinkovic, Alexander Zimmerer, Felix Zimmermann

**Affiliations:** 1 Arcus Sportklinik, Pforzheim, Germany; 2 Department of Trauma Surgery, Orthopaedics, and Plastic Surgery, University Medicine Göttingen, Göttingen, Germany; 3 Center for Musculoskeletal Surgery, Charité-Universitaetsmedizin Berlin, Campus Mitte, Charitéplatz 1, 10117 Berlin, Germany; 4 Berufsgenossenschaftliche Unfallklinik Ludwigshafen, Ludwig-Guttmann-Straße 13, 67071 Ludwigshafen am Rhein, Germany

**Keywords:** Patellar instability, Quality of life, SF-36, BPII 2.0

## Abstract

**Purpose:**

There is a paucity of quality of life (QoL) assessments in studies evaluating patients treated for recurrent lateral patellar dislocation (LPD). The primary aim of this study was to investigate whether mental well-being is impaired in patients with chronic (recurrent) LPD and, if so, to assess whether the mental health-related QoL dimension improves equivalently to the physical-related QoL dimension after successful surgical treatment.

**Methods:**

Thirty-eight patients with recurrent LPD over a mean course of the disease of 4.7 ± 3.9 years (1—18 years) prior to surgery were included. Generic health-related QoL (HRQoL) (Short Form 36; SF-36) and disease-specific QoL (Banff Patella Instability Instrument 2.0; BPII 2.0) were assessed preoperatively and after a mean follow-up of 3.5 ± 0.8 years (2 – 5 years) postoperatively.

**Results:**

Untreated LPD significantly impacted the physical dimension of patients’ generic HRQoL and their disease-specific QoL. When compared to age-equivalent normative data sets, the mental HRQoL dimension was not reduced prior to operative treatment but increased during the follow-up period. Surgical treatment normalized the physical dimension of patients’ generic HRQoL and significantly improved their disease-specific QoL. However, BPII 2.0 values remained reduced, albeit patellae were successfully stabilized.

**Conclusion:**

The results of this study indicate that patients with recurrent LPD are generally in good mental health, although physical impairment is striking. Notwithstanding that surgery prevented further dislocations and normalized the generic HRQoL, the disease-specific QoL remained reduced as far as this can be interpreted without population-based data.

**Level of evidence:**

Level IV; Retrospective case series.

## Introduction

Lateral dislocation of the patella (LPD) is one of the most common knee joint injuries in adolescents and young adults [9, 28]. The overall risk for experiencing a second LPD after primary dislocation is 29% to 37% [19, 29] and increases to 54% after 15 years [27]. Studies have consistently reported that the younger the patient and the more risk factors are present, the higher the risk of experiencing a second dislocation [2]. Although patient-reported outcomes improve after conservative treatment, knee joint function typically does not return to normal, and chronic (recurrent) LPD affects knee joint function as much as anterior cruciate ligament (ACL) deficiency [23, 30]. However, surgical treatment for LPD is still reluctant [30], leading to reduced physical activity in sports and daily life [21], with possible implications on patients’ health-related quality of life (HRQoL) [25].

Without appropriate treatment, LPD can persist for years and relevantly impacts patients’ disease-specific quality of life (QoL) [11, 12, 22]. However, HRQoL includes not only the area of ​​physical well-being but also mental well-being. As a long-lasting disease, LPD might negatively affect all dimensions of HRQoL, but only limited information is available on the influence of LPD on the mental QoL dimension [3, 4]. Therefore, the aim of this study was (1) to investigate whether mental well-being is impaired in patients with recurrent LPD and, if so, (2) to assess whether the mental HRQoL dimension improves equivalently to the physical HRQoL dimension after successful surgical treatment. The hypothesis was that both the physical and mental dimensions of HRQoL are reduced in patients with LPD prior to operative treatment.

## Methods

This study received approval from the ethics committee in Baden-Württemberg, Germany (F-2019–070), and informed consent was obtained from each patient. A priori power analysis (G*Power; Version 3.1.3) revealed that a minimum of 34 patients were needed to detect a difference in PCS and MCS score values of 5 points (SD 10) with a power of 0.80 (alpha error = 0.05, effect size d = 0.5). Therefore, a total of 38 randomly selected patients (m/f 13/25; age 20 ± 5 years (14 – 33 years)) who underwent primary surgical treatment for recurrent LPD between April 2015 and April 2018 (out of 122 patients (m/f 43/79; age 21.5 ± 6 years (14 – 40 years)) were included in this retrospective analysis of a longitudinally maintained database. The included patients had at least 2 patellar dislocations (23 patients [61%] had ≥ 3 dislocations) over a mean course of the disease of 4.7 ± 3.9 years (1—18 years) prior to surgery. The demographics and anatomical risk factor profile of the study group are listed in Table 1. In all patients, surgical treatment included reconstruction of the medial patellofemoral ligament (MPFL-R). MPFL-R was combined with a tibial tubercle osteotomy in 17 patients, with a femoral varization osteotomy in 1 patient, and with a torsional osteotomy at the distal femur in 2 patients according to previously published thresholds [33].

**Table 1:** Demographics and anatomical risk factors of patellar instability in the study cohort

	Study cohort (*n* = 38)
Male/female	13/25
Age	20 ± 5 years (14 – 33 years)
Follow-up	3.5 ± 0.8 years (2 – 5 years)
Mean course of the disease	4.7 ± 3.9 years (1—18 years)
Trochlear dysplasia	
None	8 (21.1%)
Typ A	14 (36.8%)
Typ B	10 (26.3%)
Typ C	6 (15.8%)
Typ D	-
TT–TG distance (mm)	13.6 ± 5.0
TT–PCL distance (mm)	23.7 ± 3.2
Patellar height	1,2 ± 0,3
( +) Varus / (-) Valgus (°)	- 0,9 ± 3.0

Trochlear dysplasia was assessed according to Dejour’ classification and patellar height was assessed according to the Caton-Deschamps Index. Shown are the mean values ± standard deviations and absolute and relative frequencies. *TT–TG* tibial tuberosity–trochlear groove, *TT–PCL* tibial tuberosity–posterior cruciate ligament

Generic HRQoL and disease-specific QoL were assessed preoperatively and after a mean follow-up of 3.5 ± 0.8 years (2 – 5 years) postoperatively using the Short Form 36 (SF-36) [5] and the Banff Patella Instability Instrument 2.0 (BPII 2.0). The patients were contacted again for postoperative data collection [3]. The SF-36 score was norm-referenced using the means and standard deviations of the US norm sample cohort from 1998. [7, 32]. The mental (MCS) and physical (PCS) health component summary scores were calculated [7] using both the orthogonal (_orth_MCS and _orth_PCS) and oblique (_obl_MCS and _obl_PCS) calculation methods according to Taft et al. and Farivar et al., respectively, to avoid artificial increases in the MCS value by single orthogonal assessment [8, 17, 31]. The results of the summary scales were compared to the German normative, age-equivalent SF-36 data set, which is 48.0 points for the MCS and 55.8 points for the PCS [7].

The exclusion criteria were as follows: (1) previous surgical patellar stabilizing procedures (soft-tissue or bony procedures), (2) patellofemoral pain without objective findings of LPD, and (3) untreated knee ligament injury or insufficiency other than LPD and previous knee joint surgery (e.g., ACL reconstruction).

### Statistics

Continuous data were assessed for normality and are presented as the mean, standard deviation (SD), and range. Unpaired and paired 2-tailed t tests, one-sample t tests, and Wilcoxon signed rank tests were used to assess differences between the pre- and postoperative clinical data and between the study group and the normative data sets. Pearson r and Spearman rank correlation coefficients were used to assess the correlation between the pre- and postoperative BPII 2.0, PCS, and MCS score values, and linear regression was used to assess correlations between BPII 2.0, PCS, and MCS values over the preoperative course of the disease. All analyses were performed using Prism (Version 4; GraphPad Software). The level of significance was set at *p* < 0.05. 5).

## Results

Preoperatively, the _orth_PCS and the _obl_PCS values were significantly reduced at 39.4 ± 12.1 and 42.1 ± 10.6 points (both *p* < 0.0001), respectively, when compared to normative data sets, and the BPII 2.0 value averaged 35.6 ± 16.4 points. The mean _obl_MCS equaled normative data (47.6 ± 10.1 points; p = 0.81), and the _orth_MCS was even slightly higher than the norm (51.9 ± 11.9 points; p = 0.048).

**Fig. 1:**
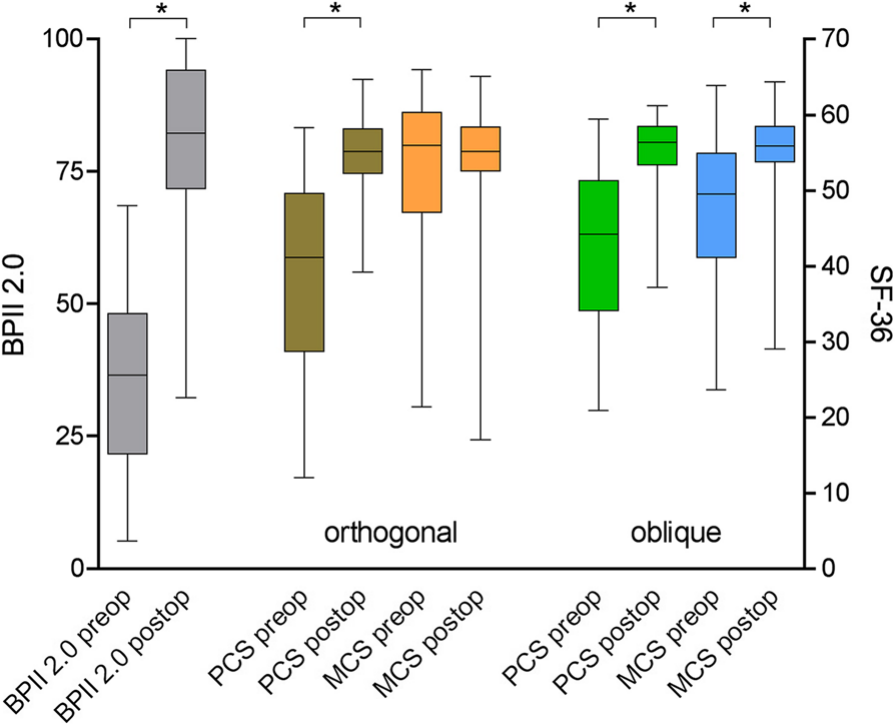
Changes in PCS, MCS, and BPII 2.0 score values from pre- to postoperatively. PCS, physical component summary score; MCS, mental component summary score; obl, oblique; orth, orthogonal; BPII 2.0, Banff Patella Instablity Instrument 2.0

Postoperatively, the _orth_PCS increased to 54.5 ± 5.2 points (*p* < 0.0001), the _obl_PCS increased to 55.3 ± 4.6 points (*p* < 0.0001), equivalent to normative PCS values (p = 0.15 and p = 0.54), and the BPII 2.0 increased significantly to 79.6 ± 17.5 points (*p* < 0.0001). The mean _orth_MCS remained unchanged at 53.4 ± 8.5 points (p = 0.52), and the _obl_MCS increased significantly to 54.3 ± 6.8 points (*p* < 0.0001) (Fig. 1).

**Fig. 2:**
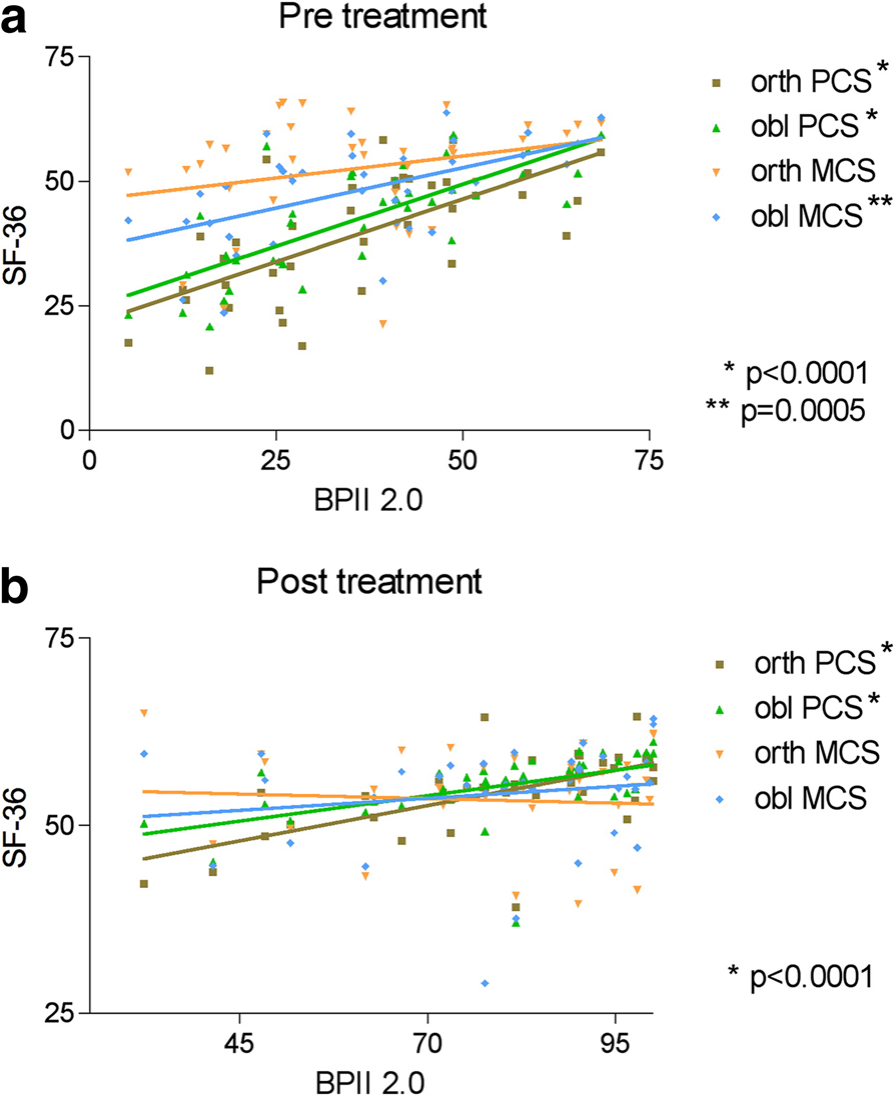
**a** and **b**: Preoperative (**a**) and postoperative (**b**) correlations between PCS, MCS, and BPII 2.0 score values. PCS, physical component summary score; MCS, mental component summary score; obl, oblique; orth, orthogonal; BPII 2.0, Banff Patella Instablity Instrument 2.0

A high correlation was found between the BPII 2.0 score and both PCS values preoperatively (_orth_PCS Pearson r = 0.66; *p* < 0.0001; _obl_PCS p = 0.75; *p* < 0.0001) and postoperatively (_orth_PCS Spearman rho = 0.63; *p* < 0.0001; _obl_PCS rho = 0.68, *p* < 0.0001) (Fig. 2a and b). The _obl_MCS was correlated with the BPII 2.0 score preoperatively (Pearson r = 0.54; P = 0.0005) but not postoperatively (Spearman rho = 0.22; p = 0.18), and the _orth_MCS values were not correlated, either pre- or postoperatively (Fig. 2a and b). In addition, no correlation was found for any PCS, MCS, or BPII 2.0 values considering the duration of the disease until treatment (all *p* > 0.05), nor were any differences noted between the sexes or between isolated and combined procedures (data not shown). No redislocation occurred in any of the patients during the follow-up period.

## Discussion

This study aimed to investigate the effect of chronic (recurrent) LPD on the physical and mental dimensions of patients’ QoL from pre- to postoperatively. The results confirmed the hypothesis that LPD significantly impacts the physical dimension of generic HRQoL and patients’ disease-specific QoL when assessed with the SF-36 and the BPII 2.0. When compared to age-equivalent normative data sets, the MCS values were not reduced prior to operative treatment, indicating an overall good mental health status of the investigated patients.

The rationales for performing patellar-stabilizing surgery include facilitating an anxiety-free return to activities of daily living and sports [21] and reducing the risk of patellofemoral osteoarthritis (OA) in the long term [26]. The success of a treatment is reflected in the HRQoL of the patient; accordingly, treatment outcomes should be measured not only by physical findings but also by the individual assessment of patients’ life satisfaction [1]. Self-administered questionnaires have been implemented to assess the generic HRQoL and the disease-specific (knee-related) QoL of patients, allowing clinicians to explore potential QoL predictors in patellar instability. The latter might help identify patient subgroups most likely to benefit from operative treatment and to identify those patients suitable for nonoperative therapy.

To date, BPII 2.0 is the only validated measure developed to assess disease-specific QoL in patients with LPD [10, 16]. The BPII 2.0 is a 23-point questionnaire covering the areas "Symptoms and Physical Complaints", "Work or School-related Problems", "Leisure Time/Sport/Activity", "Lifestyle", and "Social Life/Feelings/Emotions”. In an analysis of the responsiveness of common patient-reported outcome measures (PROM) used to assess patients with LPD, BPII 2.0 showed the highest effect size without any ceiling effects [20]. In previous studies, BPII 2.0 ranged between 24 and 38 points (maximum achievable number of points 100) prior to treatment [11, 22], underlining the relevantly reduced knee-related QoL of affected patients. The severity of patellar maltracking (J-sign), body mass index (BMI), bilateral symptoms, and age at first dislocation were significant predictors of lower BPII 2.0 scores [12, 22].

Surgical treatment resulted in a significant improvement in patients’ QoL at a mean of 3.5 years postoperatively. Although these overall findings warrant interpretation as ‘good’ results and no patellar redislocation occurred in any of the patients, BPII 2.0 score values remained approximately 20 points less than ‘normal’, given a possible maximum BPII 2.0 score value of 100 points. This indicates that despite a stable patella, reduced disease-specific QoL is still evident in certain patients. This finding supports previous findings that the presence or absence of recurrent patellar dislocation alone is not an adequate parameter to evaluate the success of a treatment [21]. Studies have reported that even after patellar-stabilizing surgery, individuals cease sport participation [24], experience ongoing subjective feelings of instability [13], and fear reinjury [15]. In addition, complex surgical interventions might expose one’s to additional physical and mental distress, which could further increase fear avoidance behaviour [18]. In this regard, postoperative knee pain and limited range of knee joint motion influenced patients’ knee-related QoL more relevantly than a postoperative redislocation of their kneecap did [34]. However, since population-based data on BPII 2.0 values are not yet available, interpretation of postoperative BPII 2.0 values remains limited.

HRQoL is a multifactorial construct that includes physical, social, emotional, and psychological components [1, 14]. Previous research recommended that when evaluating orthopedic interventions, at least one generic health status questionnaire should be included in addition to disease-specific instruments [6]. Although disease-specific measures appear more sensitive in detecting changes from pre- to postoperatively, as confirmed by the results obtained from this study, generic health measures, such as the SF-36, allow broader insight into patients’ QoL and allow comparisons across conditions and populations [6]. This study found a high correlation between the PCS and BPII 2.0 at both the preoperative and postoperative levels, indicating that BPII 2.0 measures a broad spectrum of QoL constructs [3]. This confirms previous findings by Becher et al. [3], who analysed 64 patients with patellar instability during a BPII 2.0 validation study. In addition, their study found no correlation between BPII 2.0 and the MCS and reported a mean MCS score value of 51.28 ± 9.8 points in their study population, which is comparable to the postoperative _orth_MCS and _obl_MCS score results of this study. However, MCS data calculation has been a matter of debate in recent years [17]. Since four of the eight subscales of the SF-36 have negative weights when using the orthogonal calculation method, scoring low on these subscales might raise the MCS ‘false too high’ due to impaired physical function. This might explain why the _orth_MCS in this study scored even higher than the norm, whereas the _obl_MCS scored equal to normative data.

Quality of life measures remain underreported in studies evaluating outcomes for LPD [20]. However, the need for more research in this field can be derived from a few studies that have used the Knee Osteoarthritis and Outcome Score (KOOS). Although the KOOS assesses knee-related impairments without a specific patellar instability component, it has been successfully used in previous investigations evaluating this patient group [30]. Studies have reported that the lowest values were found for the KOOS ‘Sports/Recreation’ and ‘QoL’ subscales [21, 30]. Thus, this study aimed to further contribute to the importance of QoL assessment in patients with LPD. However, the results must be interpreted under consideration of several limitations. First, only the short-term results of a small cohort of patients without a control group are presented in this study. Thus, the findings may not be representative of the treatment group as a whole, since patellar instability exhibits a broad range of patient-specific characteristics. Second, it is important to note that this study used PROMs only and that the results were not correlated with objective clinical findings, functional performance, other activity scores, or imaging. Third, normative values of the BPII 2.0 are not yet available. Consequently, a conclusive statement on treatment success when evaluated with the BPII 2.0 remains limited. Finally, the limitations and potential bias characteristics of a retrospective study need to be considered.

## Conclusion

The results of this pilot study indicate that patients with recurrent LPD are generally in good mental health, although physical impairment is striking. Notwithstanding that surgery prevented further patellar dislocations and normalized the generic HRQoL, the disease-specific QoL remained reduced as far as this can be interpreted without population-based data sets.

## Data Availability

The datasets used and/or analysed during the current study are available from the corresponding author on reasonable request.
